# Sleep, social media use and mental health in female adolescents aged 12 to 18 years old during the COVID-19 pandemic

**DOI:** 10.1186/s12887-023-04218-4

**Published:** 2023-08-14

**Authors:** Wikanda Chalermchutidej, Boonying Manaboriboon, Gornmigar Sanpawitayakul, Supparat Theppiban, Supinya In-iw

**Affiliations:** 1 Division of Ambulatory Pediatrics, Department of Pediatrics, Faculty of Medicine Siriraj Hospital, Mahidol University, Bangkok, Thailand; 2 Department of Pediatrics, Faculty of Medicine Siriraj Hospital, Mahidol University, Bangkok, Thailand

**Keywords:** Anxiety, Depression, Social media use, Sleep quality, COVID-19 lockdown

## Abstract

**Background:**

Adolescents with high social media (SM) use experienced poor sleep quality and high anxiety and depression levels. The study aimed to investigate the characteristics of sleep, use of SM, mental health in female aged 12 to 18 years old, and to assess the association between poor sleep, SM usage, and mental health.

**Methods:**

In total, 219 Thai female adolescents were recruited between December 2019 and September 2020 and completed self-administrative questionnaires three periods of time (baseline, 3 months and 6 months later). The questionnaires included: the Pittsburgh Sleep Quality Index (PSQI), depression screening (PHQ-9), Screen for Child Anxiety Related Emotional Disorders (SCARED). Demographic and use of social media data were also included. Cochran’s Q test, correlation coefficient, and binary logistic regression were performed.

**Results:**

Participants’ mean age was 14.52 (range 12–17) years. Average Thai-PSQI global scores did not differ during 3 periods (p = 0.13) but average time of sleep latency, sleep duration, and SM usage were significant different (p = 0.002, p = 0.001, and p = < 0.001, respectively). There were positive correlations between PSQI scores and total SM usage at baseline (r = 0.14; *P* < 0.05) and 6 months (r = 0.20; *P* < 0.05). Anxiety, depression, and self-perception of poor sleep were significantly related to poor sleep quality during the 3 periods. After adjusting for confounding factors, depression and self-reported poor sleep were the only significant factors predicting poor sleep quality.

**Conclusions:**

Poor sleep was associated with SM usage, depression, and anxiety in this population. Time-limited SM usage should be implemented for Thai female adolescents to improve sleep-related outcomes.

## Introduction

Good sleep quality was associated with improved attention, behavior, learning, memory, emotional regulation, quality of life, and mental and physical health [1]. In contrast, inadequate sleep had negative health consequences, for example, obesity, diabetes, hypertension, and depression [2, 3]. Furthermore, inadequate sleep for adolescents and adults has been shown to carry behavioral risks such as self-harm and suicide attempts [4–6]. The recommended duration of sleep for adolescents is 8 to 10 h/day [7]. In Thailand, the average sleep period among adolescents was 7 h a day which was lower than the recommendation [8]. Research in the USA found that a duration of more than 9 h/day compensated for a debt of sleep and could reduce fatigue [9]. Those with sleep debt, which defined as not getting adequate sleep over a series of days, needed more hours for sleep compensation. However, the risk for insulin resistance and metabolic syndrome due to sleep deficiency is not minimized after oversleeping [10].

Changes in sleep–wake regulation and circadian rhythm during adolescence were biological process which might contribute to sleep problems in adolescents [11]. Studies from South Korea, Northern Taiwan, and Germany showed that the duration of sleep among adolescents decreased with increasing age [12–14]. Furthermore, many factors were also contributed to insufficient sleep during this stage such as caffeine consumption, academic performance, and the use of electronic media [15]. Thai adolescents used social media (SM) for recreational purposes and the most preferred social platforms were Facebook, YouTube, Line, and Twitter [16]. The prevalence of Facebook addiction among Thai high school students was higher than those in Philippine, Germany, and Turkey [17]. Other researches found that SM use was strongly associated with sleep disturbance, depression, and anxiety [18, 19]. The previous studies also demonstrated that adolescents who had higher levels of SM use for emotional investment, those giving value of spending online time to make them feel happy or connect them to the past memories or other activities, both in general and at night, experienced poorer sleep quality, lower self-esteem, and higher levels of anxiety and depression [20, 21]. Nonetheless, those did not explore factors associated with SM use and sleep quality, such as anxiety, depression, or years of schooling. The previous study showed the linear association between multiple platform of social media use for personal purposes and emotional well-being such as stress and poor sleep quality [16]. The strongest predictor of having very poor sleep quality was Twitter-addiction [16]. Furthermore, risk of depression among adolescents increased whenever increasing use of SM [22].

The systematic review and meta-analysis regarding the effect of the COVID-19 pandemic on sleep quality in adolescents found that sleep quality worsened during those pandemic, despite of increasing sleep duration [23]. Also, sleep characteristics such as increased sleep duration, late bedtimes, and poor sleep quality were related to the changes in bedtime routines and screen time usage during those pandemic [23]. Furthermore, time spent on social media had a stronger relationship with emotional distress among female rather than male. This previous study also proposes gender differences in the relationship between SM use and well-being which demonstrated that female who interacted with SM higher at age 10 were associated with declines in well-being [24]. Similar to the study during the COVID-19 outbreak in China, it found that being female, more stress, and spending over 60 min on SM were associated with anxiety symptoms increment [25].

Even though most studies were done during that pandemic, but the study period was quite limited. The longitudinal studies exploring the association between SM use and sleep quality for different time periods were required. Furthermore, during the COVID-19 pandemic, the majority of adolescents stayed at home with social isolation. Factors related to sleep, psychological well-being and SM use in this outbreak was interesting to explore its association. The current study aimed to investigate the characteristics of sleep, use of social media (and electronic devices) and mental health in the study population during COVID-19 lockdown period and after school re-opening. The secondary objective was to assess the association between poor sleep quality and use of social media, and mental health.

## Methods

### Study design

This prospective cohort study was conducted between December 2019 and September 2020. Before starting this research, its protocol was approved by the IRB in our institution. It was conducted at the University hospital. All methods were performed in accordance with the relevant guidelines and regulations.

### Participants

The recruitment process was carried out at a private all-girl school in Bangkok, Thailand. The school was selected because it exemplified a focus population that represented a gender-specific pattern of SM use. Participants were female adolescents (aged 12 to 18 years) who studied in grades 7 through 12 and had agreed to participate. Informed consents were obtained from their parents or legal guardians.

### Data collection

A total of 219 participants completed a range of self-administered electronic questionnaires at baseline, 3 months, and 6 months. Exclusion criteria were having underlying diseases, especially obstructive sleep apnea and mental health illnesses. Only 6 participants did not complete all questionnaires and were excluded from data analysis. Data collection was carried out during 2 semesters and during the intervening school break. The baseline was the month of the second-semester final examinations (February 2020). The 3-month follow-up occurred during the COVID-19 pandemic and associated lockdown period (May 2020). The 6-month follow-up was at the beginning of the first semester after the school reopening with new normal school policy (September 2020). The self-administered questionnaires consisted of a health behavior survey, an SM usage questionnaire, the Pittsburgh Sleep Quality Index (PSQI) Instruments, the Thai version of the Patient Health Questionnaire (PHQ-9), and the Thai version of the Screen for Child Anxiety Related Emotional Disorders (SCARED) questionnaire.

### Materials

All previous tools in this study were validated for adolescent population and used under owners’ permission.

The health behavior survey collected information about age, grade point average (GPA), learning problems, body satisfaction, exercise, caffeine consumption, and risky behaviors. This questionnaire was routinely used to assess the adolescent’s behaviors particularly risky behaviors [26].

We developed the SM use questionnaires based on the objective of this study and it was subjectively reported by answering a specially prepared questionnaire. It had been content-validated by 3 specialists in adolescent medicine. The questionnaire focused on the types of SM, the time spent, the devices used to access SM, the main purpose of use, and problems related to SM usage such as feeling distressed if not using SM, being told by family and friends to decrease using SM, using SM to relax or to socialize, resulting in sleep insufficiency, desiring to stop using SM and seeking help.

Sleep quality was evaluated with the PSQI-Thai version. This 19-item self-administered questionnaire has a sensitivity of 77.78% and a specificity of 93.33% [27]. PSQI was validated for the adolescent population [28]. It assesses sleep quality over the previous month. Scores are obtained for 7 domains: subjective sleep quality, sleep latency, sleep duration, habitual sleep efficiency, sleep disturbances, use of sleep medications, and daytime dysfunction. A total score of greater than 5 (range 0–21) is classified as poor sleep quality.

The Thai version of the Screen for Child Anxiety Related Emotional Disorders (SCARED) is a validated questionnaire for anxiety screening. Permission to use was obtained from Ularntinon S [29]. This 41-item rating scale questionnaire used a 3-point scale (0 = almost never, 1 = sometimes, and 2 = often). Total SCARED scores ≥ 25 were likely to signify an anxiety disorder. Total scores ≥ 9 (from items 5, 7, 14, 21, 23, 28, 33, 35, and 37) indicated the possibility to be a generalized anxiety disorder. Total scores ≥ 8 (from items 3, 10, 26, 32, 39, 40, and 41) suggest to be a social anxiety disorder.

Depression was identified using the Thai version of PHQ-9 [30], validated by Kongsook et al. [31] This tool was recommended as part of the Thai Clinical Practice Guideline for depression to assess all Thai adolescents during routine health check-up. The self-report instrument uses 9 questions to assess the symptoms of depression experienced by an individual during the previous 2 weeks. All questions are scored from 0 (not at all) to 3 (nearly every day). A total score ≥ 7 were the optimal cut-off point for diagnosing depression in the Thais, with a sensitivity of 86.15% and specificity of 83.12%.

### Statistical analysis

Statistical analyses were performed using PASW Statistics for Windows (version 18; SPSS Inc., Chicago, IL, USA). The characteristics of the samples were summarized using frequency, percentage, mean, and standard deviations for normally distributed variables. A generalized linear model (using repeated measures) was performed to compare continuous variables during the 3 periods, and Cochran’s Q test was performed to identify the differences in the values of the 3 periods. The correlation coefficient was used to analyze the association between PSQI scores and depression, anxiety, and related factors. Univariate and multivariate analyses were performed using binary logistic regression. The dependent variable was sleep quality, while the independent variables were the total duration of SM use, the SCARED-Thai version total scores (anxiety disorder), generalized anxiety disorder scores, the social anxiety disorder scores, and the depression scores. Unadjusted and adjusted odds ratios with their 95% confidence intervals were reported. All significant factors from the multivariate analysis were used to put in the second analysis for adjusted odd ratios. A *p* value of less than 0.05 was considered statistically significant.

## Results

Demographic data are summarized in Table 1. The mean age was 14.52 (± 1.59) years, and the mean GPA was 3.24 (range 1.45–4.00). Half of the students consumed caffeine after lunch (54.8%) and felt depressed (55.7%). A total of 81.3% reported that their sleep quality was poor.

**Table 1:** Demographic data (N = 219)

		Number (%)
Mean age (years)(SD)		14.52(1.59)
Learning problems	None Attention deficit Mathematics, reading, writing problems Others	73(33.3) 83(37.9) 53(24.2) 10(4.6)
Skipping meals		215(98.2)
Dissatisfaction with body weight		136(62.1)
Regular exercise (5 days/week)		48(21.9)
Regular drinks	Tea and coffee Juice Soft drink Others	77(34.1) 58(26.3) 57(25.9) 14(6.3)
Caffeine consumption after lunch		120(54.8)
Ever had substance use	No Alcohol consumption Others	180(81.8) 21(9.5) 19(8.7)
Depression	No Depressed Depressed with suicidal ideation	97(44.3) 82(37.4) 40(18.3)
Ever had sexual intercourse		4(1.8)
Reported of poor sleep quality		178(81.3)
Reported sleep-related problems		157 (71.7)
Reason for delaying going to bed	None Social media usage Sleep- related problems Others	42(19.2) 26(11.9) 133(60.7) 18(8.2)
Average GPA (mean)(range)		3.24(1.45-4.00)

Table 2 showed the characteristics of the SM use, PSQI, depression, and anxiety at baseline, 3 months, and 6 months period. The 3 periods had a statistically significant difference in the average SM use during their respective survey week. The most common SM platforms were YouTube, Line, and Instagram. The proportion of participants who played online games during the school break was higher than the corresponding levels for the first and second semesters (42.9%, 29.7%, and 32%, respectively; *p* = 0.002). A total of 154 students (70.2%) used a smartphone to access SM at baseline, which was significantly higher than the values for the 2 other periods (p < 0.001). The majority of the students used SM after 4 PM on weekdays (78.1%, 90.9%, and 83.3%) and after 6 PM on weekends (79.9%, 65.7%, and 76.7%). Most of the students used SM to relax, with the highest level of use for this purpose during the school break (84%, 94.5%, and 89%; *p* = 0.003). The average duration of sleep (hours/night) was 6.50 at baseline, 6.99 at 3 months, and 6.64 at 6 months (p = 0.001). At these points, only a certain amount of students (8.7%, 13.2%, and 5.5%) reported sleeping for more than 8 h (*p* = 0.02). At baseline, 56.1% were diagnosed with poor sleep quality, and the proportion decreased at 3 months- (53.4%) and 6-months follow-up (50.7%). At 6-months-period, a significant number of students reported feeling sad for more than 2 weeks. The level was higher than those of the other 2 periods (*p* < 0.001), similar to the average self-rated happiness score (*p* < 0.001). There were no significant differences in anxiety among those during the 3 periods.

**Table 2:** Characteristics of social media usage, Pittsburgh Sleep Quality Index, depression, and anxiety related disorder among high school female adolescents (N = 219)

Characteristics		N(%)			*P* value
		At base line	At 3 months	At 6 months	
**Social media usage**					
Average social media usage (hours/day) (SD)		7.14(3.95)	8.18(4.12)	8.01(3.52)	< 0.001
Average frequency of using during past week (days) (SD)		6.65(1.14)	6.69(0.95)	6.83(0.65)	0.04
Social media usage	YouTube Line Instagram Twitter Facebook Thai-weblog	204(93.2) 196(89.5) 178(81.3) 148(67.6) 76(34.7) 108(49.3)	196(89.5) 177(80.8) 194(89.5) 160(73.1) 80(36.5) 86(39.3)	195(89) 181(82.6) 194(88.6) 160(73.1) 77(35.2) 52(23.7)	0.19 0.81 0.90 0.09 0.85 < 0.001
Online gaming		70(32)	94(42.9)	65(29.7)	0.002
Device	Smartphone Computer/tablet/smartphone	154(70.2) 65(29.8)	67(30.6) 152(69.4)	86(39.3) 133(60.7)	< 0.001 < 0001
Having problems with social media usage		72(32.9)	36(16.4)	42(19.2)	< 0.001
Using social media at weekdays	6.01 A.M.-5.59 PM 6.00 PM-12.00 PM 0.01-6.00 AM	35(16) 160(73.1) 11(5)	20(9.1) 197(90) 2(0.9)	36(16.4) 176(81.3) 5(2.3)	0.02 0.001 0.03
Using social media at weekend	6.01 A.M.-5.059 PM 6.00 PM-12.00 PM 0.01-6.00 AM	40(18.3) 141(64.4) 34(15.5)	75(34.2) 122(55.7) 22(10)	51(23.3) 151(68.9) 17(7.8)	< 0.001 0.004 0.006
Feeling distressed if not using social media		91(41.6)	92(42)	94(42.9)	0.99
Family and friends tell you to decrease using social media		146(66.7)	140(63.9)	122(55.7)	0.01
Using social media to relax		184(84)	207(94.5)	195(89)	0.003
Using social media to socialize		108(49.3)	126(57.5)	107(48.9)	0.02
Social media causes sleep insufficiency		137(62.6)	102(46.6)	109(49.9)	< 0.001
Desired to stop using social media and seek help		30(13.7 )	21(9.6)	19(8.7)	0.07
**Pittsburgh Sleep Quality Index**					
Poor Thai-PSQI global scores		123(56.1)	117(53.4)	111(50.7)	0.08
Average Thai-PSQI global scores for sleep quality (SD)		6.29 (2.71)	6.13 (2.67)	5.91(2.65)	0.13
Average sleep latency (minutes)		19.83(21.25)	15.60(14.06)	16.38(18.02)	0.002
Average sleep duration (hours/night)		6.50(1.79)	6.99(1.78)	6.64(1.38)	0.001
Self-perception of poor sleep quality		188(85.8)	187(85.4)	183(83.6)	0.45
Sleep latency (minutes)	≤ 15 16–30 31–60 > 60	103(47) 80(36.5) 20(9.1) 4(1.8)	124(56.6) 79(36.1) 14(6.4) 1(0.5)	125(57.5) 74(33.8) 11(5) 4(1.8)	0.01 0.33 0.06 0.32
Sleep duration	< 6 h 6–8 h > 8 h	47(18.7) 145(66.2) 19(8.7)	41(18.7) 149(68.0) 29(13.2)	38(17.4) 168(76.7) 12(5.5)	0.27 0.04 0.02
Habitual sleep efficiency	> 85% 75–84% 65–74% < 65%	24(11.0) 124(56.6) 56(25.6) 10(4.6)	29(13.2) 120(54.8) 59(26.9) 11(5.0๗	28(12.8) 115(52.5) 68(31.1) 7(3.2)	0.85 0.59 0.46 0.54
Sleep disturbance (sum scores)	0 1–9 10–18 19–27	14(6.4) 162(74.0) 43(19.6) 0	12(5.5) 159(72.6) 48(21.9) 0	15(6.9) 168(76.7) 36(16.4) 0	0.83 0.38 0.18 -
Use of sleep medication		21(9.6)	12(5.5)	21(9.6)	0.02
Daytime dysfunction		100(45.7)	111(50.7)	122(55.7)	0.92
**Depression**					
Average self-rating happiness score (SD)		7.31(1.9)	7.47(1.9)	6.94(2)	< 0.001
Average PHQ-9^*#*^–Thai version scores (SD)		7.78(5.17)	7.17(5.6)	6.89(5.7)	< 0.001
PHQ-9^*#*^-Thai version scores	None (0–6) Mild (7–12) Moderate to severe (13–27)	112(51.1) 72(32.9) 35(16.0)	127(58.0) 64(29.2) 28(12.8)	118(53.9) 70(32.0) 31(14.2)	0.07 0.50 0.31
Feeling sad for more than two weeks		44(20.1)	31(14.2)	55(25.1)	< 0.001
Ever had suicidal ideation		40(18.3)	50(22.8)	42(19.2)	0.34
Ever had suicidal plan		21(9.6)	27(12.3)	18(8.2)	0.15
**Anxiety-related disorders**					
Average SCARED^*##*^-Thai version scores (SD)		29.68(13.07)	30.19(15.90)	30.54(14.29)	0.58
SCARED^*##*^-Thai version (scores ≥ 25)		142(64.8)	135(61.6)	149(68.0)	0.16
Generalize anxiety disorder (scores ≥ 9)		120(54.8)	118(53.9)	124(56.6)	0.72
Social anxiety disorder scores (scores ≥ 8)		91(41.6)	83(37.9)	88(40.2)	0.55

^*#*^PHQ-9 : Patient health questionaires-9 ^*##*^SCARED: Screen for Child Anxiety Related Emotional Disorders

The association between PSQI scores and the total duration of SM usage during the 3 periods, showed a statistically significant positive correlation only at the beginning of the study (r = 0.14; *p* < 0.05; Table 3). However, there was a significant positive correlation between PSQI scores and anxiety, depression, self-perception of poor sleep quality, and self-reported learning problems. Academic performance, caffeine consumption, and risky behaviors were not associated with poor sleep quality.

**Table 3:** Association between Pittsburg Sleep Quality Index scores and social media usage, anxiety, depression, learning, and risky behaviors (N = 219)

	PSQI scores		
	At baseline	At 3 months	At 6 months
Total duration of social media usage	0.14^*^	0.08	0.20^*^
Average SCARED^*#*^-Thai version scores	0.21*	0.33**	0.36**
Generalized anxiety disorder scores	0.24**	0.37**	0.35**
Social anxiety disorder scores	0.08	0.18*	0.19*
Self-rated happiness scores	-0.34**	-0.44**	-0.52**
Average PHQ-9-Thai version scores	0.43**	0.49**	0.55**
Self-perception of poor sleep quality	0.29**	0.30**	0.33**
Grade point Average	-0.08	-0.06	-0.08
Self-reported learning problems	0.20*	0.18*	0.13*
Having risky behaviors	0.10	0.18*	0.11

^*#*^PHQ-9 : Patient health questionnaires-9 ^*##*^SCARED: Screen for Child Anxiety Related Emotional Disorders

*P value < 0.05; **P value < 0.001

Factors associated with poor sleep quality are listed in Table 4. SM use was associated with poor sleep quality only at 6-months follow-up (odds ratio, 1.11; 95% CI, 1.02–1.22). Students who had higher total SCARED scores, generalized anxiety disorder scores, and social anxiety disorder scores were more likely to have poor sleep quality (Table 4). The self-perception of poor sleep quality was associated with poor sleep quality in all 3 periods (4.59 [95% CI, 2.23–9.44]; 2.83 [95% CI, 1.39–5.72]; and 6.44 [95% CI, 3.09–13.42]). After adjusting for the significant factors in our univariate analysis, a multivariate analysis found that only depression and self-perception of poor sleep quality were associated with poor sleep quality at baseline and 6-month follow-up.

**Table 4:** Factors associated with poor Pittsburg Sleep Quality Index scores (N = 219)

	Poor PSQI scores at baseline		Poor PSQI scores at 3 months		Poor PSQI scores at 6 months	
	OR	aOR	OR	aOR*	OR	aOR*
Total duration of social media usage	1.07(0.98–1.16)		1.05(0.97–1.12)		1.11(1.02–1.22)*	1.06(0.96–1.18)
SCARED^*##*^-Thai version scores	1.05 (1.01–1.07)*	1.02(0.95–1.09)	1.06(1.03–1.08)**	0.96(0.91–1.02)	1.06(1.03–1.08)**	1.02(0.96–1.09)
Generalized anxiety disorder scores	1.12(1.04–1.19)*	1.00(0.86–1.17)	1.21(1.13–1.31)**	1.21(1.06–1.39)*	1.17(1.09–1.25)**	1.03(0.89–1.21)
Social anxiety disorder scores	1.13(1.03–1.24)*	1.07(0.92–1.25)	1.15(1.06–1.25)*	1.04(0.91–1.18)	1.10(1.02–1.20)*	0.96(0.83-1,0.12)
PHQ-9^*#*^-Thai version scores	1.2(1.10–1.31)**	1.13(1.01–1.25) *	1.27(1.15–1.39)**	1.20(1.07–1.33)*	1.29(1.17–1.40)**	1.22(1.09–1.35)**
Grade Point Average	0.77(0.43–1.36)		1.22(0.71–2.01)		0.84(0.49–1.46)	
Self-reported learning problems	2.18(1.17–4.03)*	1.29(0.63–2.65)	1.94(1.07–3.53)*	1.47(0.74–2.89)	1.29(0.71–2.34)	
Having risky behaviors	1.17(0.63–2.13)		1.49(0.83–2.70)		1.05(0.59–1.87)	
Self-perception of poor sleep quality	4.59(2.23–9.44)**	3.79(1.73–8.34) *	2.83(1.39–5.72)*	1.86(0.85–2.05)	6.44(3.09–13.42)**	5.89(2.55–13.59)**

^*#*^PHQ-9 : Patient health questionnaires-9 ^*##*^SCARED: Screen for Child Anxiety Related Emotional Disorders

*P value < 0.05; **P value < 0.001

aOR : adjusting for the significant factors in univariate analysis

## Discussion

During the lockdown female adolescents reported an increase in sleep problems; mainly long sleep latency and total scores of PSQI, used more electronic devices and social media. However, differences in the frequency of anxiety and depression were not significant between the three periods. Poor sleep quality was correlated with social media use after the lockdown, but not during the lockdown, probably due to the flexibility of school schedules. Our results were similar to previous studies, showing that SM use was associated with poor sleep quality [20, 32, 33]. A systematic review found that the vast majority of studies demonstrated that screen-based media consumption, delayed bedtimes, and reduced total sleep duration resulted in adverse sleep [34]. Furthermore, poor sleep quality was associated with a higher likelihood of addiction to SM or online gaming [16, 35]. Interestingly, our study revealed that poor sleep quality was not correlated with SM use during the school break. The explanation for this might be that even though the students had a greater opportunity to sleep longer during the school break than during the semesters, the total duration of sleep was still below the recommended level, resulting in no difference in sleep quality scores. However, the data revealed that the proportion of students who slept for more than 8 h was higher during the school break than during the two semesters. A review of the literature related to school start times and sleep among adolescents also showed that delaying school start time increased sleep duration by delaying a wakening time [36].

The COVID-19 pandemic and lockdown in Thailand could have inadvertently encouraged students to access SM due to the move to online learning, virtually with computers and mobile devices, making them more access to SM. Our study found that the average usage of SM and online gaming during the lockdown period (i.e., the break between the two semesters) were greater than that during the two semesters, when the students were back to school. Moreover, the use of SM on weekends and weekdays during the school break differed from the use during the first and second semesters. Students did not access SM before school start time because they tended to wake up late. This contrasted with the second-semester final examination period, when the proportion of SM users increased significantly after midnight both weekdays and weekends.

The findings of this study showed that depression and anxiety were related to poor sleep quality. This corresponded with many studies that demonstrated an association between SM use and mental health outcomes [19, 21]. The COVID-19 pandemic and lockdown did not affect negative emotions in our study population.In contrast, previous studies on anxiety and depression during COVID-19 lockdowns showed that students experienced more stress, anxiety, and depression [37–39].

The strength of the study was a longitudinal study exploring the association between SM use and sleep quality for three different time period. The study results were similar to prior studies which showed that adolescents who use SM tended to have a poorer sleep quality, and higher levels of anxiety and depression [19, 20].

The limitations of the current study must be considered. First, the population in the study is not generalizable because it might not imply male gender and public schools. Second, the study did not have the control group. Third, as the study was questionnaire-based, it reported the subjective assessments of the students. Therefore, the accuracy and reliability of the information they provided in their responses may be compromised, given that the information was based on their recall or individual assessments. Finally, self-reported responses about emotions may not align with the clinical diagnoses of mental health specialists. Further experimental and observational research were needed to investigate details for both the female and male populations, which could clarify the association between SM usage and sleep quality.

## Conclusions

Female adolescents used more electronic devices and social media during the COVID-19 lockdown and reported an increase in sleep problems. However, between the three periods of time, there was no difference in the frequency of anxiety and depression. Poor sleep quality was correlated with social media use after the lockdown, but not during the lockdown. Higher levels of SM usage were associated with poorer sleep quality and psychological distress. The health guidance of time-limited use of SM, increasing duration of sleep, and non-SM activities participation should be implemented for Thai students to prevent sleep-related problems.

## Data Availability

The datasets used and analyzed during the current study available from the corresponding author on reasonable request.

## References

[1] Paruthi S, Brooks LJ, D’Ambrosio C, Hall WA, Kotagal S, Lloyd RM (2016). Recommended amount of Sleep for Pediatric populations: a Consensus Statement of the American Academy of Sleep Medicine. J Clin Sleep Med.

[2] Cappuccio FP, Miller MA (2017). Sleep and Cardio-Metabolic Disease. Curr Cardiol Rep.

[3] Itani O, Jike M, Watanabe N, Kaneita Y (2017). Short sleep duration and health outcomes: a systematic review, meta-analysis, and meta-regression. Sleep Med.

[4] Liu X (2004). Sleep and adolescent suicidal behavior. Sleep.

[5] Blasco-Fontecilla H, Alegria AA, Lopez-Castroman J, Legido-Gil T, Saiz-Ruiz J, de Leon J (2011). Short self-reported sleep duration and suicidal behavior: a cross-sectional study. J Affect Disord.

[6] Gangwisch JE, Babiss LA, Malaspina D, Turner JB, Zammit GK, Posner K (2010). Earlier parental set bedtimes as a protective factor against depression and suicidal ideation. Sleep.

[7] Watson NF, Badr MS, Belenky G, Bliwise DL, Buxton OM, Buysse D (2015). Recommended amount of Sleep for a healthy adult: a Joint Consensus Statement of the American Academy of Sleep Medicine and Sleep Research Society. Sleep.

[8] Hounnaklang N, Lertmaharit S, Lohsoonthorn V, Rattananupong T (1970). Prevalence of poor sleep quality and its correlates among high school students in Bangkok, Thailand. J Health Res.

[9] Mantua J, Skeiky L, Prindle N, Trach S, Doty TJ, Balkin TJ (2019). Sleep extension reduces fatigue in healthy, normally-sleeping young adults. Sleep Sci.

[10] Koren D, Levitt Katz LE, Brar PC, Gallagher PR, Berkowitz RI, Brooks LJ (2011). Sleep architecture and glucose and insulin homeostasis in obese adolescents. Diabetes Care.

[11] Owens J (2014). Insufficient sleep in adolescents and young adults: an update on causes and consequences. Pediatrics.

[12] Huang YS, Wang CH, Guilleminault C (2010). An epidemiologic study of sleep problems among adolescents in North Taiwan. Sleep Med.

[13] Yang CK, Kim JK, Patel SR, Lee JH (2005). Age-related changes in sleep/wake patterns among korean teenagers. Pediatrics.

[14] Loessl B, Valerius G, Kopasz M, Hornyak M, Riemann D, Voderholzer U (2008). Are adolescents chronically sleep-deprived? An investigation of sleep habits of adolescents in the Southwest of Germany. Child Care Health Dev.

[15] Ahrberg K, Dresler M, Niedermaier S, Steiger A, Genzel L (2012). The interaction between sleep quality and academic performance. J Psychiatr Res.

[16] Abu-Snieneh HM, Aroury AMA, Alsharari AF, Al-Ghabeesh SH, Esaileh AA (2020). Relationship between sleep quality, using social media platforms, and academic performance among university students. Perspect Psychiatr Care.

[17] Khumsri J, Yingyeun R, Mereerat M, Hanprathet N, Phanasathit M (2015). Prevalence of Facebook Addiction and related factors among Thai High School Students. J Med Assoc Thai.

[18] Levenson JC, Shensa A, Sidani JE, Colditz JB, Primack BA (2016). The association between social media use and sleep disturbance among young adults. Prev Med.

[19] Primack BA, Escobar-Viera CG (2017). Social media as it interfaces with Psychosocial Development and Mental Illness in Transitional Age Youth. Child Adolesc Psychiatr Clin N Am.

[20] Woods HC, Scott H, #Sleepyteens (2016). Social media use in adolescence is associated with poor sleep quality, anxiety, depression and low self-esteem. J Adolesc.

[21] Lin LY, Sidani JE, Shensa A, Radovic A, Miller E, Colditz JB (2016). Association between Social Media Use and Depression among U.S. Young adults. Depress Anxiety.

[22] Liu M, Kamper-DeMarco KE, Zhang J, Xiao J, Dong D, Xue P. Time spent on Social Media and Risk of Depression in Adolescents: a dose-response Meta-analysis. Int J Environ Res Public Health. 2022;19(9). 10.3390/ijerph19095164 PMC9103874 35564559

[23] Richter SA, Ferraz-Rodrigues C, Schilling LB, Camargo NF, Nunes ML (2023). Effects of the COVID-19 pandemic on sleep quality in children and adolescents: a systematic review and meta-analysis. J Sleep Res.

[24] Booker CL, Kelly YJ, Sacker A (2018). Gender differences in the associations between age trends of social media interaction and well-being among 10–15 year olds in the UK. BMC Public Health.

[25] Hou F, Bi F, Jiao R, Luo D, Song K (2020). Gender differences of depression and anxiety among social media users during the COVID-19 outbreak in China:a cross-sectional study. BMC Public Health.

[26] Ruangkanchanasetr S, Plitponkarnpim A, Hetrakul P, Kongsakon R (2005). Youth risk behavior survey: Bangkok, Thailand. J Adolesc Health.

[27] Sitasuwan T, Bussaratid S, Ruttanaumpawan P, Chotinaiwattarakul W (2014). Reliability and validity of the Thai version of the Pittsburgh Sleep Quality Index. J Med Association Thailand = Chotmaihet thangphaet.

[28] Piboonworakulkij R, Kongsaktrakul C, Santati S (2019). Factors Predicting Sleep Quality of secondary School students in Bangkok. Thail JNSCU.

[29] Birmaher B, Brent DA, Chiappetta L, Bridge J, Monga S, Baugher M (1999). Psychometric properties of the screen for child anxiety related Emotional Disorders (SCARED): a replication study. J Am Acad Child Adolesc Psychiatry.

[30] Lotrakul M, Sumrithe S, Saipanish R (2008). Reliability and validity of the Thai version of the PHQ-9. BMC Psychiatry.

[31] Kongsook T, Janthong S, Prukkanone B, Sukhawaha S, Leejongpermpoon J, Aroonpaisarn S (2018). Criterion-Related validity of the 9 questions Depression Rating Scale revised for Thai Central Dialect. J Psychiatr Assoc Thailand.

[32] Aldhawyan AF, Alfaraj AA, Elyahia SA, Alshehri SZ, Alghamdi AA (2020). Determinants of subjective poor sleep quality in social media users among freshman College Students. Nat Sci Sleep.

[33] Mohammadbeigi A, Absari R, Valizadeh F, Saadati M, Sharifimoghadam S, Ahmadi A (2016). Sleep quality in medical students; the impact of Over-Use of Mobile cell-phone and Social Networks. J Res Health Sci.

[34] LeBourgeois MK, Hale L, Chang A-M, Akacem LD, Montgomery-Downs HE, Buxton OM (2017). Digital Media and Sleep in Childhood and Adolescence. Pediatrics.

[35] Wong HY, Mo HY, Potenza MN, Chan MNM, Lau WM, Chui TK et al. Relationships between Severity of Internet Gaming Disorder, Severity of Problematic Social Media Use, Sleep Quality and Psychological Distress. Int J Environ Res Public Health. 2020;17(6). 10.3390/ijerph17061879 PMC7143464 32183188

[36] Wheaton AG, Chapman DP, Croft JB (2016). School Start Times, Sleep, behavioral, Health, and academic outcomes: a review of the literature. J Sch Health.

[37] Rehman U, Shahnawaz MG, Khan NH, Kharshiing KD, Khursheed M, Gupta K et al. Depression, anxiety and stress among Indians in Times of Covid-19 Lockdown. Community Ment Health J. 2020:1–7. 10.1007/s10597-020-00664-x PMC7309680 32577997

[38] Odriozola-González P, Planchuelo-Gómez Á, Irurtia MJ, de Luis-García R (2020). Psychological effects of the COVID-19 outbreak and lockdown among students and workers of a spanish university. Psychiatry Res.

[39] Khan AH, Sultana MS, Hossain S, Hasan MT, Ahmed HU, Sikder MT (2020). The impact of COVID-19 pandemic on mental health & wellbeing among home-quarantined bangladeshi students: a cross-sectional pilot study. J Affect Disord.

